# An Unusual Cause of Digit Pain: Acrometastasis From Lung Adenocarcinoma

**DOI:** 10.7759/cureus.38475

**Published:** 2023-05-03

**Authors:** Noelle Provenzano, Lindsey Forker, Emerson T Trimble, Mimi Lyang, Mark Morginstin

**Affiliations:** 1 Internal Medicine, Einstein Medical Center Montgomery, East Norriton, USA; 2 Family Medicine, Touro College of Osteopathic Medicine, New York, USA; 3 Surgery, Touro University California, Vallejo, USA; 4 Hematology, Oncology, Einstein Medical Center Montgomery, East Norriton, USA

**Keywords:** finger metastasis, adenocarcinoma, bone metastasis, lung cancer, acrometastasis

## Abstract

Acrometastasis accounts for 0.1% of all cases of metastatic cancer, with the most common primary tumor being lung cancer. Since acrometastasis is extremely rare and it generally has a nonspecific clinical presentation, it provides a diagnostic dilemma. We present a case of a 70-year-old female with a painful swollen right index finger which was found to be a metastatic lesion from adenocarcinoma of the lung. The patient expired within one month of diagnosis due to complications from her rapidly progressive metastatic cancer.

## Introduction

Lung cancer is the leading cause of cancer-associated deaths worldwide. Adenocarcinoma of the lung is the most common type of lung cancer seen in the United States. It is strongly associated with smoking and falls under the category of non-small cell lung cancer [1]. Acrometastasis, metastases distal to the elbow and knee, accounts for only 0.1% of all cases of metastatic cancer [2]. The most common primary tumor site associated with acrometastasis is the lung, followed by colon, breast, and genito-urinary tract cancers [3]. Metastatic lesions in the finger are more often associated with lung cancer, while lesions in the toes are associated with genito-urinary malignancies. The distal phalanx, especially the thumb, is the most common site for hand metastasis [4]. Acrometastasis is mainly found in patients with widespread disease and less commonly as an initial sign of cancer; however, in some cases, it is the first indication of a late presenting malignancy. Acrometastasis is most commonly treated with amputation. The prognosis of acrometastatic cancer after diagnosis has a mean survival time of less than six months [3].

Metastasis to the hand is rare for any cancer, especially as a sign of recurrent cancer. We report a case of a 70-year-old female considered in remission from adenocarcinoma of the lung whose recurrence was noted by a metastatic lesion to her finger. This case was presented as a poster at the 2021 ACP Southeastern Regional Posters Day and Doctors Dilemma in Philadelphia, Pennsylvania, on October 20, 2021.

## Case presentation

A 70-year-old female with a past medical history of chronic bronchitis, asthma, hypertension, hyperlipidemia, venous insufficiency, and tobacco use (76-pack-year history, quit 16 years prior) presented with a right index finger pain without any known trauma to an outpatient primary care provider office.

Five months prior, the patient was diagnosed with primary lung cancer with a 2.1 x 1.1 mm right middle lobe adenocarcinoma stage cT1c N0 M0. At that time, a positive emission tomography (PET) scan showed no local regional lymphadenopathy or distant metastases. She was treated with definitive stereotactic body radiation therapy to the nodule, as she was not a surgical candidate due to medical comorbidities. She was considered in remission post-treatment.

**Figure 1:**
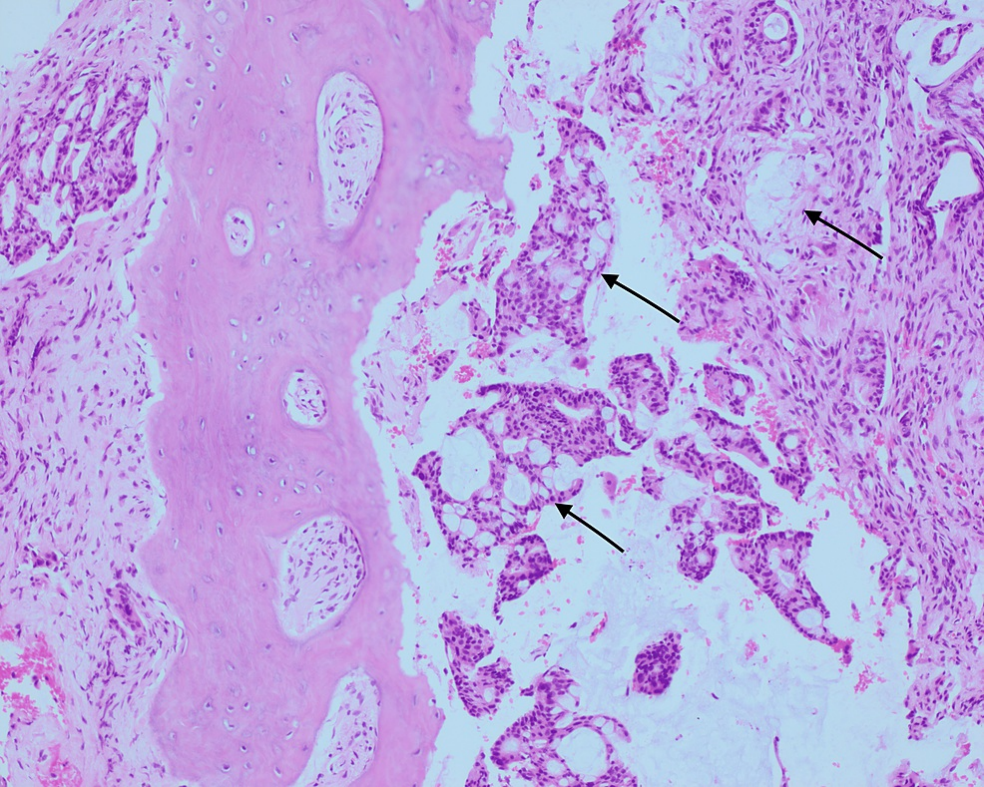
100x hematoxylin and eosin stain. Demonstrating metastatic adenocarcinoma displaying glandular and cribriform patterns of growth. The neoplastic cells demonstrate mucinous cytoplasmic vacuoles

**Figure 2:**
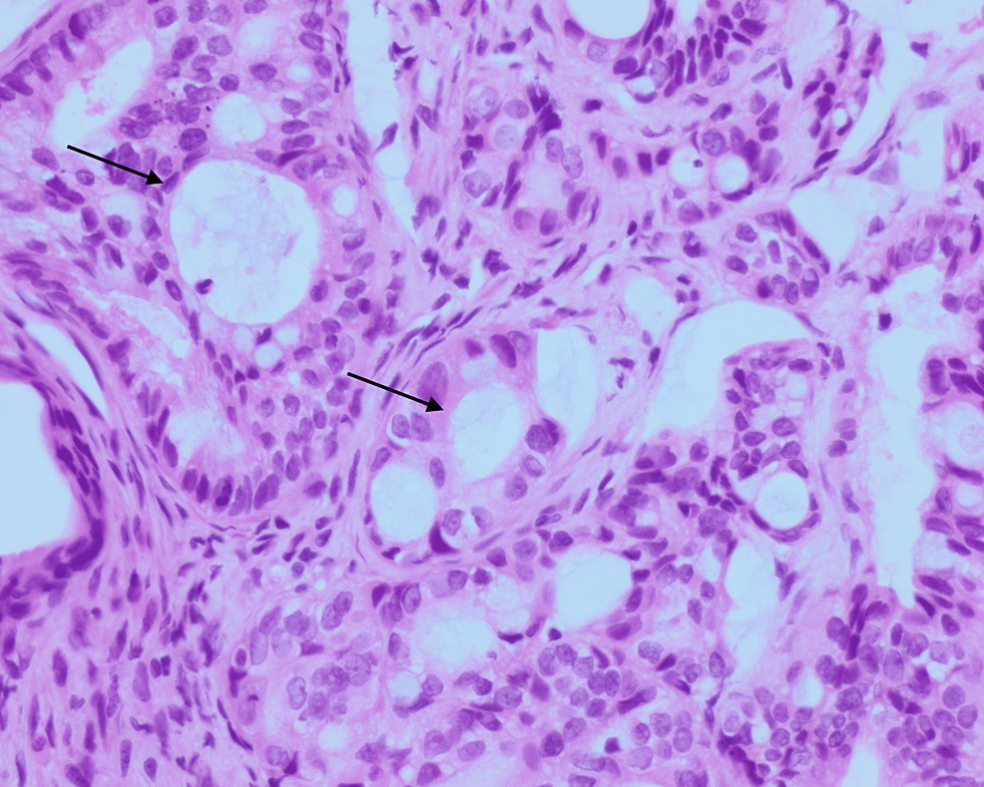
400x hematoxylin and eosin stain. Demonstrating metastatic adenocarcinoma. Neoplastic cells demonstrate moderate nuclear pleomorphism and cytoplasmic vacuolization

In the current presentation, the patient expressed a six-week history of right index finger pain. The initial workup included plain radiograph films that demonstrated a lytic lesion of the right proximal phalanx of the second finger, which was worse on the radial side. Initial concern for infection versus malignancy led to a biopsy, which demonstrated metastatic adenocarcinoma displaying glandular and cribriform patterns of growth. The neoplastic cells demonstrate mucinous cytoplasmic vacuoles (Figures 1, 2).

**Figure 3:**
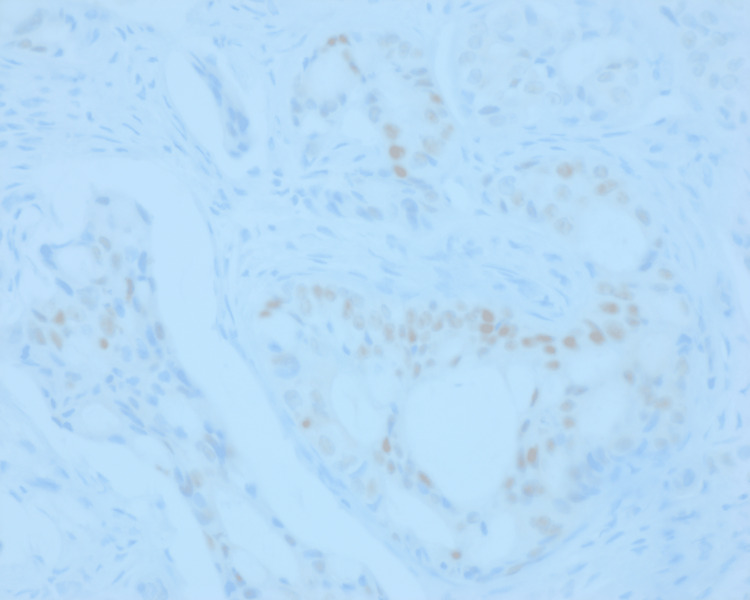
400x CDX2 IHC. Neoplastic cells demonstrate focal nuclear positivity for CDX2. The CDX2 positivity is clearly seen scattered throughout the figure

CDX2 immunohistochemistry (IHC) stain demonstrated focal nuclear positivity for CDX2 (Figure 3).

**Figure 4:**
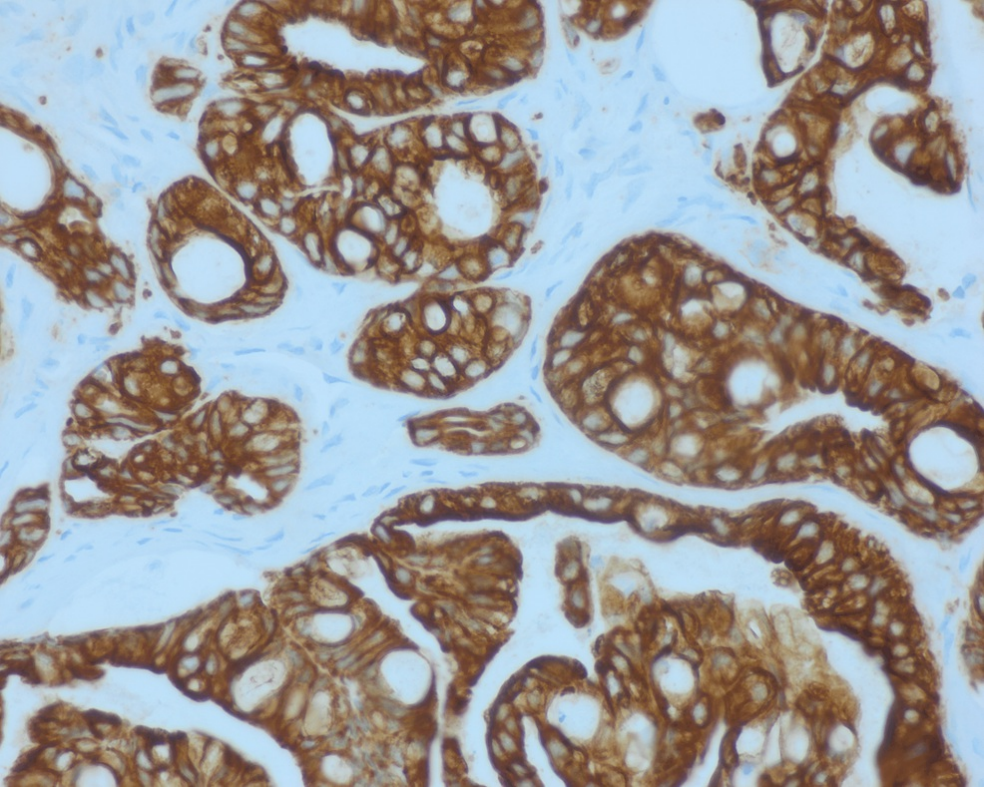
400x CK7 IHC. Neoplastic cells demonstrate uniform strong cytoplasmic positivity for cytokeratin CK7; the uptake is clearly seen throughout the whole figure

With CK7 IHC, the neoplastic cells demonstrated strong cytoplasmic positivity for cytokeratin CK7 (Figure 4).

The pathology was consistent with metastatic adenocarcinoma, likely originating from the lung. The patient was scheduled for repeat imaging and oncology follow-up.

Shortly after her biopsy resulted, the patient developed back pain. She was evaluated by orthopedic surgery and subsequently completed an X-ray of her lumbosacral spine which demonstrated multilevel degenerative disease of the lumbosacral spine but no evidence of osseous metastases. One month later, before she could follow up with her oncologist, the patient was admitted to the hospital due to hypoxia and continued back pain. CT scan revealed diffuse osteolytic metastasis involving the lumbar vertebral bodies, pathologic compression fractures of T10 and T11 vertebral bodies, and possible epidural involvement along with pneumonia and a right pleural effusion. CT scan of her brain revealed lytic lesions of occipital condyles, left-sided upper ribs, and right medial clavicle consistent with osseous metastases. Unfortunately, the patient expired during her admission due to hypoxic hypercapnic respiratory failure.

## Discussion

Although the patient’s cancer was initially localized to the lung and underwent definite treatment, her cancer recurrence was identified due to metastasis to her finger. This patient’s presentation is rare as she was found to have a metastatic lesion in her finger, while imaging of her spine did not show involvement. Generally, acrometastasis is found in patients with widespread disseminated disease. It was not until a month later that the patient developed osseous metastases in her spine.

Acrometastasis can manifest as a silent tumor presenting as inflammatory lesions that can be mistaken for osteomyelitis, cysts, gout, ganglia, tuberculous dactylitis, pyogenic granuloma, and primary skin tumors [4]. Originally described by Handley in 1906, acrometastasis is an unusual location for bony metastasis as osseous metastasis generally occurs in red marrow-rich areas such as vertebral bodies [5]. It is generally accepted that acrometastasis occurs through circulatory spread which explains why lung cancer accounts for the vast majority of cases. Other solid organ tumors have to go through the hepatic or pulmonary capillary beds before reaching the extremities and will get trapped [6].

There are several theories regarding the pathophysiology of acrometastasis. Increased blood flow to the area supports the phenomena of metastasis being more common in the dominant hand [7]. Repetitive trauma may make the surrounding tissue less resistant to tumor emboli that then settle and grow in the skeletal muscle [7]. Another thought is that repeated trauma may induce prostaglandins, which serve as chemotactic factors and encourage cell migration and adherence [8]. It has also been hypothesized that metastatic hand lesions occur more often in the dominant hand [6]. This hypothesis is consistent with our case as well.

In addition to the sites of metastatic lesions, histopathology also plays a role in the prognosis of adenocarcinoma. The cribriform growth pattern in lung adenocarcinoma is more aggressive than the acinar pattern. The cribriform pattern shows a higher mitotic figure and larger areas of necrosis. It is associated with increased lymphatic/vascular invasion, mucin production, and a higher pathological TNM stage [9]. This patient demonstrated the cribiform growth pattern which may have contributed to her poor outcome.

Treatment of acrometastasis encompasses several different modalities, aiming to treat the current disease and prevent the local recurrence of diseases. Amputation is generally the first choice of treatment, especially in diseases limited to the distal phalanges [6]. Lesions involving the joints are not suitable for amputation, as amputation would lead to significant functional impairment. Second-line therapy is radiation because it increases the risk of pathologic fractures [10]. Another second-line treatment is polymethylmethacrylate bone cement which helps to fill in the lesion and provide structural support [11]. Wen-Chih et al. describe a case in which the patient was treated with an intralesional excisional biopsy and then filled the defect with an artificial bone graft, which was successful at alleviating the patient’s pain and preserving function [12]. Given the incredibly poor prognosis, oftentimes this is treated with palliative radiation. While treatment options may be limited by the time acrometastasis is diagnosed, it is important to recognize it earlier for patients who may benefit from amputation.

## Conclusions

Although extremely rare, acrometastasis should be considered in patients with a smoking history who present with digit pain, even in the absence of an underlying malignancy. Our case demonstrates that high clinical suspicion is needed in order to expedite treatment. This also demonstrates the importance of history taking in forming a differential.
